# Optimizing care coordination to address social determinants of health needs for dual-use veterans

**DOI:** 10.1186/s12913-021-07408-x

**Published:** 2022-01-12

**Authors:** Heidi Sjoberg, Wenhui Liu, Carly Rohs, Roman A Ayele, Marina McCreight, Ashlea Mayberry, Catherine Battaglia

**Affiliations:** 1 Department of Veterans Affairs, Eastern Colorado Health Care System, 1700 N. Wheeling St, Aurora, CO 80045 USA; 2 Anschutz Medical Campus Colorado School of Public, University of Colorado, 13001 E. 17th Pl, Aurora, CO 80045 USA

**Keywords:** social determinants of health, veterans, social work, emergency departments, veterans health administration

## Abstract

**Background:**

Veterans increasingly utilize both the Veteran’s Health Administration (VA) and non-VA hospitals (dual-users). Dual-users are at increased risk of fragmented care and adverse outcomes and often do not receive necessary follow-up care addressing social determinants of health (SDOH). We developed a Veteran-informed social worker-led Advanced Care Coordination (ACC) program to decrease fragmented care and provide longitudinal care coordination addressing SDOH for dual-users accessing non-VA emergency departments (EDs) in two communities.

**Methods:**

ACC had four core components: 1. Notification from non-VA ED providers of Veterans’ ED visit; 2. ACC social worker completed a comprehensive assessment with the Veteran to identify SDOH needs; 3. Clinical intervention addressing SDOH up to 90 days post-ED discharge; and 4. Warm hand-off to Veteran’s VA primary care team. Data was documented in our program database. We performed propensity matching between a control group and ACC participants between 4/10/2018 – 4/1/2020 (*N*- = 161). A joint survival model using Markov Chain Monte Carlo technique was employed for 30-day outcomes. We performed Difference-In-Difference analyses on number of ED visits, admissions, and primary care physician (PCP) visits 120-day pre/post discharge.

**Results:**

When compared to a matched control group ACC had significantly lower risk of 30-day ED visits (Hazard Ratio (HR) = 0.61, 95% Confidence Interval (CI) = (0.42, 0.92)) and a higher probability of PCP visits at 13–30 days post-ED visit (HR = 1.5, 95% CI = (1.01, 2.22)). Veterans enrolled in ACC were connected to VA PCP visits (50%), VA benefits (19%), home health care (10%), mental health and substance use treatment (7%), transportation (7%), financial assistance (5%), and homeless resources (2%).

**Conclusion:**

We developed and implemented a program addressing dual-users’ SDOH needs post non-VA ED discharge.

Social workers connected dual-users to needed follow-up care and resources which reduced fragmentation and adverse outcomes.

**Supplementary Information:**

The online version contains supplementary material available at 10.1186/s12913-021-07408-x.

## Background

### Problem Description

The Veterans Health Administration’s (VA) Maintaining Systems and Strengthening Integrated Outside Networks (MISSION) Act increased Veterans’ ability to access non-VA hospital care, improving Veterans’ access to care [1, 2] and increasing dual-use. Collaboration between VA and non-VA hospitals is often complex and fragmented [3–5] causing adverse outcomes and leaving Veterans to manage their own care coordination [6] or rely on VA primary care clinics who are often not informed of Veterans’ non-VA hospital visits [7]. Effective care coordination addressing social determinants of health (SDOH) for dual-use Veterans to avoid adverse outcomes is essential [3–5, 8, 9]. SDOH include where people live, work, health, access to health care, economic stability, education and social and community contexts [10]. SDOH contribute significantly to biopsychosocial wellbeing and are often associated with emergency department (ED) use [11, 12]. Dual-use Veterans have complex SDOH needs, including difficulty accessing health care due to barriers navigating the VA system, not receiving VA benefits (e.g. financial, VA enrollment to access health care, etc.), financial strain and access to housing, psychosocial stressors and functional limitations, making self-managed care coordination challenging [13, 14]. Dual-use Veterans are at higher risk of experiencing adverse outcomes [4, 5] including increased 30-day hospital readmissions [4, 15], conflicting treatments and duplicated tests [16–18], medication errors [19–22], and decreased satisfaction with their care [23]. Care coordination programs may decrease these adverse outcomes, enhance care and address SDOH for dual-use Veterans by linking them to essential social and medical resources.

Veterans are most vulnerable during transition periods [3, 24] (e.g., from hospital to home) and often need care coordination. We developed quality improvement (QI) nurse-led Community Hospital Transitions Program (CHTP) to enhance care coordination for Veterans hospitalized at non-VA hospitals and transitioning back home [25, 26]. While implementing CHTP, we learned Veterans who accessed non-VA EDs were not receiving necessary follow-up care since there was no structured care coordination processes in place between non-VA EDs and VA(27). Thus, we met with the Eastern Colorado Health Care System (ECHCS) Veteran Research Engagement Board (VREB) to receive guidance and consultation on strategies to address this. Through ongoing meetings with the VREB we developed the social worker-led QI Advanced Care Coordination (ACC) program [27] to address Veterans’ SDOH post non-VA ED visits.

### Rationale

Haggerty’s Continuity of Care (CoC) Framework [28] guided our understanding of the VA’s care coordination gaps and informed our development of ACC to follow an ideal care transitions process. The CoC Framework has 3 types of continuity: 1) Informational which uses information from previous events (e.g., hospitalization records) to inform an individual’s care; 2) Management to support consistent methods to manage an individual’s health conditions; and 3) Relational to provide an ongoing relationship between the individual and providers [28]. ACC encompassed all three domains to promote successful implementation. Additionally, ACC was founded in the following evidence-based key components recommended for effective care coordination programs for Veterans [29–31]: 1) Complete a biopsychosocial and functional comprehensive needs assessment; 2) Enroll Veterans into the program during periods of transition; 3) Frequent phone calls and in-person visits; and 4) Utilize a multidisciplinary approach to navigate the Veteran’s needs.

### Specific Aims

The purpose of this manuscript is to describe the ACC program and disseminate the results. ACC had three specific aims: **Aim 1**: Develop and implement a comprehensive care coordination intervention for dual-use Veterans to address SDOH during care transitions. **Aim 2**: Disseminate to a second VA. **Aim 3**: Develop a toolkit and training materials to facilitate dissemination of the intervention to other VAs.

## Methods

### Context

Informed by a pre-implementation assessment of the current transitions of care processes [32], and supported by existing evidence-based practices, we developed ACC to address a fragmented care coordination process. Patient perspectives on care coordination needs were paramount to the development and implementation of ACC. To ensure patient needs were addressed effectively we met with the VREB to obtain guidance. The VREB is comprised of eight Veterans, three VA employees who serve as liaisons to the board and represent two VA research centers, and one non-VA employee to facilitate meetings. Established in 2014, this board provides a forum for Veterans to meet monthly to review and share their unique perspectives with researchers on proposals/interventions. Prior to meeting with the VREB we provided information on our intervention, our goals for the meeting, the program aims, and relevant education materials (Additional file 1). We reviewed our intervention ideas with the board. They advised us on ways to further develop and improve our intervention and program materials to better meet dual-use Veterans’ needs and have a patient-centered approach. For example, they requested we educate Veterans on how to access their Patient Aligned Care Team (PACT) social worker as most Veterans do not know this is an available resource to them. This education was integrated into the clinical intervention component of ACC. Additionally, they revised the wording and format of our Veteran Care Card (see Intervention for details of this card) to make it more user friendly for Veterans.

### Intervention

We developed and implemented ACC (Aim 1), a social worker-led care coordination intervention that provided Veterans who accessed non-VA EDs with longitudinal case management addressing SDOH up to 90-days post ED discharge to home. Prior to ACC there was no standardized care coordination process between VA and non-VA EDs. Veterans who accessed non-VA EDs were often not linked to necessary follow-up care upon discharge to home; thus, ACC was developed and implemented to address gaps in care for dual-use Veterans and to standardize care coordination between VA and non-VA EDs. We had one full-time and one part-time social worker at ECHCS and one full-time social worker at Nebraska-Western Iowa Health Care System (NWIHCS). These social workers were hired for this role and had to be willing to conduct home and community visits. The ACC social workers had ongoing collaboration with non-VA hospital staff. Based on information from our pre-implementation assessment [32], we learned coordinating care with the VA is frustrating and challenging. Non-VA hospital staff needed to have streamlined processes with the VA to coordinate Veterans care; thus, they were motivated to partner with us. Non-VA EDs are often overburdened with the number of Veterans accessing their ED and experience challenges in contacting the VA for reimbursements and coordinating follow-up VA care for Veterans. Regular in-services with non-VA EDs enhanced stakeholder buy-in and provided education on how ACC addressed SDOH and coordinated care for Veterans with the intention of decreasing frequent utilization of non-VA EDs. Non-VA ED staff were enthusiastic to partner with us as we provided a direct contact at the VA to assist with notifying appropriate VA departments to cover Veterans’ ED visit costs, helped with coordinating VA follow-up care, and assisted with developing the Veterans’ discharge plan.Non-VA ED staff were asked to notify the ACC social workers when a Veteran visited their ED to ensure smooth care transitions post-discharge. Early notification was crucial for timely care coordination. Following this ***initial notification***, the ACC social workers reviewed charts to determine program eligibility. The ACC social workers had access to VA charts through the VA’s electronic health record system and to non-VA charts through Joint Legacy Viewer which is an established electronic health record sharing system between hospitals. No releases of information were required to access these records as Health Insurance Portability and Accountability Act (HIPAA) regulations state hospitals can disclose protected health information without patient consent or authorization for the purposes of care coordination [33].

The ACC social workers called eligible Veterans within 24 h post non-VA ED discharge to complete the ***social work comprehensive assessment*** (Additional file 2) to determine SDOH needs and develop a patient-centered care plan. This assessment took 30–60 min to complete and consisted of 21 questions pertaining to reason for referral, demographics, medical and mental health, social supports, living arrangement, education and employment, income and finances, current mental status, and psychosocial problems. Based upon this assessment, the ACC social workers utilized clinical judgement to determine the Veteran’s acuity level, with level 1 needing less case management support and level 4 needing the most. Veterans with acuity levels 1–2 were enrolled 1–4 weeks. Veterans with acuity levels 3–4 were enrolled up to 90-days post-ED discharge. Case management through phone calls was provided to all acuity levels. Home visits were completed for acuity levels 3–4 and community visits were utilized for Veterans experiencing homelessness.

Following the assessment, the ACC social workers provided ***individualized clinical interventions*** through phone calls and home/community visits. The ACC social workers continuously assessed Veterans for SDOH needs and linked them to appropriate VA and non-VA resources. All ACC participants had SDOH needs. SDOH were addressed by assisting with benefits acquisition (e.g. completing applications and/or placing referrals for financial and housing assistance and Medicaid, enrolling Veterans into VA services, etc.), providing education on accessing health care (e.g. mental health and substance use treatment, primary care appointments, etc.), scheduling health care appointments and placing treatment referrals, and addressing financial barriers (e.g. linkage to transportation resources including VA, Medicaid, and Medicare transportation, etc.). Enrolled Veterans preferences and needs informed clinical decisions and care coordination. The ACC social workers employed Motivational Interviewing techniques and teach-back methodology throughout the intervention. We developed a Veteran Care Card with information about ACC and the Veteran’s VA primary care physician (PCP) for Veterans to show to non-VA ED staff when they accessed their services to enhance care coordination between the VA and non-VA hospitals. Veterans were mailed this card either when they completed their participation in ACC (acuity levels 1–2) or during the first week of enrollment (acuity levels 3–4). The ACC social workers utilized the VA Office of Community Care (VA OCC) Care Coordination guidelines to inform the intervention [34].When the Veteran reached the 90-day point or was no longer in need of ACC care coordination, the ACC social workers completed a ***warm hand-off*** through closed loop electronic communication to the Veterans’ VA primary care team. Veterans who needed case management after 90-days were connected to their assigned VA PACT social worker. PACT social workers collaborate with the Veteran’s VA PCP to enhance care coordination and patient-centered care. Throughout ACC implementation the ACC social workers documented data in our program database.

### Setting and Participants

ACC was initially implemented in ECHCS and then disseminated to NWIHCS (Aim 2). We selected partner non-VA hospitals in Denver, Colorado and Omaha, Nebraska based on the high volume of Veterans served by these facilities. They were informed about ACC prior to launch. Veterans discharged home from non-VA EDs were referred to ACC between April 2018 to April 2020. Veterans already receiving case management in the VA were excluded from ACC to not duplicate services.

### Implementation/Evaluation Team

Our multidisciplinary team consisted of social workers, nurses, a national training educator, clinical intervention specialists and consultants, experts in qualitative and quantitative research, statistics, data management, implementation science, and health economics. Our team developed and disseminated a toolkit outlining ACC’s core components, note templates, resource guides, care coordination processes, and VA and non-VA staff and provider training materials (Aim 3). We standardized training to ensure ACC was implemented with fidelity at ECHCS and NWIHCS.

### Study of the Intervention

We evaluated the effectiveness of our intervention by comparing outcomes between ACC and a control group. Following the standard process for propensity matching, we performed propensity matching for Veterans who completed all four core components between 4/10/2018 – 4/1/2020 (*N*- = 161) before outcome comparison due to differences in patient conditions and sample sizes between ACC and control group. Control group were pulled from Veterans who had non-VA ED visits and discharged home using both Corporate Data Warehouse Fee Basis Claims System and community care Program Integrity Tools System.

To ensure standardized program delivery, the ACC social workers were trained using evidence-based training curriculum developed as part of program implementation. Fidelity to the intervention was assessed using the following methods. First, virtual learning collaborative meetings were conducted by an objective facilitator who was a social worker familiar with ACC but not involved in day-to-day ACC operations to discuss program progress, assess enrollment goals, and set benchmarks to improve outcomes. During these virtual learning collaboratives, the facilitator assessed the progress on implementing and delivery of the core components, ensuring the fidelity was maintained. Additionally, any adaptations to the program core components and program delivery were discussed and tracked using a real-time tracker and process maps. Second, the program database was designed to collect data on completion of and fidelity to program core components and to flag incomplete Veteran entries. Incomplete entries were addressed during team meetings to understand why the entries were incomplete, and whether the core components were being implemented with fidelity. Data quality reports were reviewed weekly and any discrepancies and data issues were discussed during weekly check-ins with the implementation team and the ACC social workers. Finally, we conducted site visits during mid-implementation to assess program delivery process in real-time. Each site visit included meetings to obtain feedback about the program progress from various stakeholders and real-time observations of the program staff.

### Measures

The primary outcomes were 30-day ED visits, 30-day hospital readmissions, and 30-day VA PCP visits following ED discharge. The secondary outcomes were 14-day PCP visits, 90-day ED visits and 90-day hospital readmissions. Additionally, we utilized our program database to collect data and understand what resources enrolled Veterans were linked to addressing SDOH. This database was designed for ACC, with built-in visual dashboards that enabled the ACC social workers and implementation team to track multiple points of health information of each Veteran and ACC programmatic information in real time.

### Analysis

Due to huge differences in patient conditions and sample sizes between the ACC and control groups, we performed propensity matching prior to outcome comparison. The control group were matched to ACC intervention group with exact matching on site, discharge time, race, Urban, Rural or Highly Rural, Elixhauser variables (Coagulopathy and Pulmonary Circulation Disorder) (e.g. a group of control subjects were selected as a match to the intervention subject based on having the same values on exact matching variables), and nearest neighbor matching on age, sex, all other Elixhauser comorbidity variables, and number of hospitalizations/ED visits/PCP visits in the past year. To reduce the impact of COVID-19 on our outcome comparison, we matched the control and ACC groups on the time of discharge, by quarter for patient discharged before 2020 and by month for patients discharged in 2020. We matched with a ratio of 3 control to 1 ACC patient due to the large control group sample size. The matched cohort was checked by assessing the propensity score balance between control and ACC groups, as well as the standardized differences of the matched variables. Standardized differences less than 0.1(10%) between control and treatment groups are commonly considered as negligible imbalance. After matching, all standardized differences between ACC and control groups were below 0.08 for predictive covariates, indicating appropriate covariate balance. The histogram plot of the propensity score distribution also showed well balance between two groups after matching.

To account the correlations between 30-day hospital readmissions and 30-day ED visits, we fitted these two outcomes with a joint survival model, using Markov Chain Monte Carlo technique, with 10,000 iterations and burn-in of 500. The survival models were assumed to have proportional hazard with baseline risk function of Weibull distribution. All 90-day outcomes and PCP visit outcomes were fitted with Cox proportional hazard model. The covariates included in the survival model were Elixhauser score and number of ED/hospitalization/PCP visits in the past year. Based on Kaplan Meier curves, the effect of the ACC intervention on 30-day PCP visits changed over time. We included interaction term of intervention and time in the Cox model for this outcome. To compare changes in outcomes before and after intervention, between the control and ACC intervention groups, we performed Difference-In-Difference (DID) analyses on number of ED visits, admissions, and PCP visits 120-day pre/post discharge. The DID were implemented as an interaction term between time and intervention group in a regression model for each outcome.

### Ethical Considerations

ACC is a Department of Veterans Affairs grant funded QI program (see Ethics approval and consent to participate). We were exempt from the Institutional Review Board. Appropriate regulatory approvals were obtained to implement this program.

## Results

From 4/10/2018—4/1/2020 we received 1,605 referrals from non-VA EDs. Of those, 79% were ineligible to be enrolled in ACC because they did not meet inclusion criteria. Reasons for ineligibility included: 47% were hospitalized/admitted to an inpatient facility (e.g., Skilled Nursing Facilities), 17% had confirmed VA case management (e.g., established spinal cord injury patients), 3% lived outside geographical regions served by ECHCS and NWIHCS, 0.60% were dangerous to staff and 0.40% were readmitted to the ED. The following of the 79% were excluded for the following reasons: 8% declined VA care and 5% died. There were 19% who were lost to follow-up post referral to ACC.

**Figure 1:**
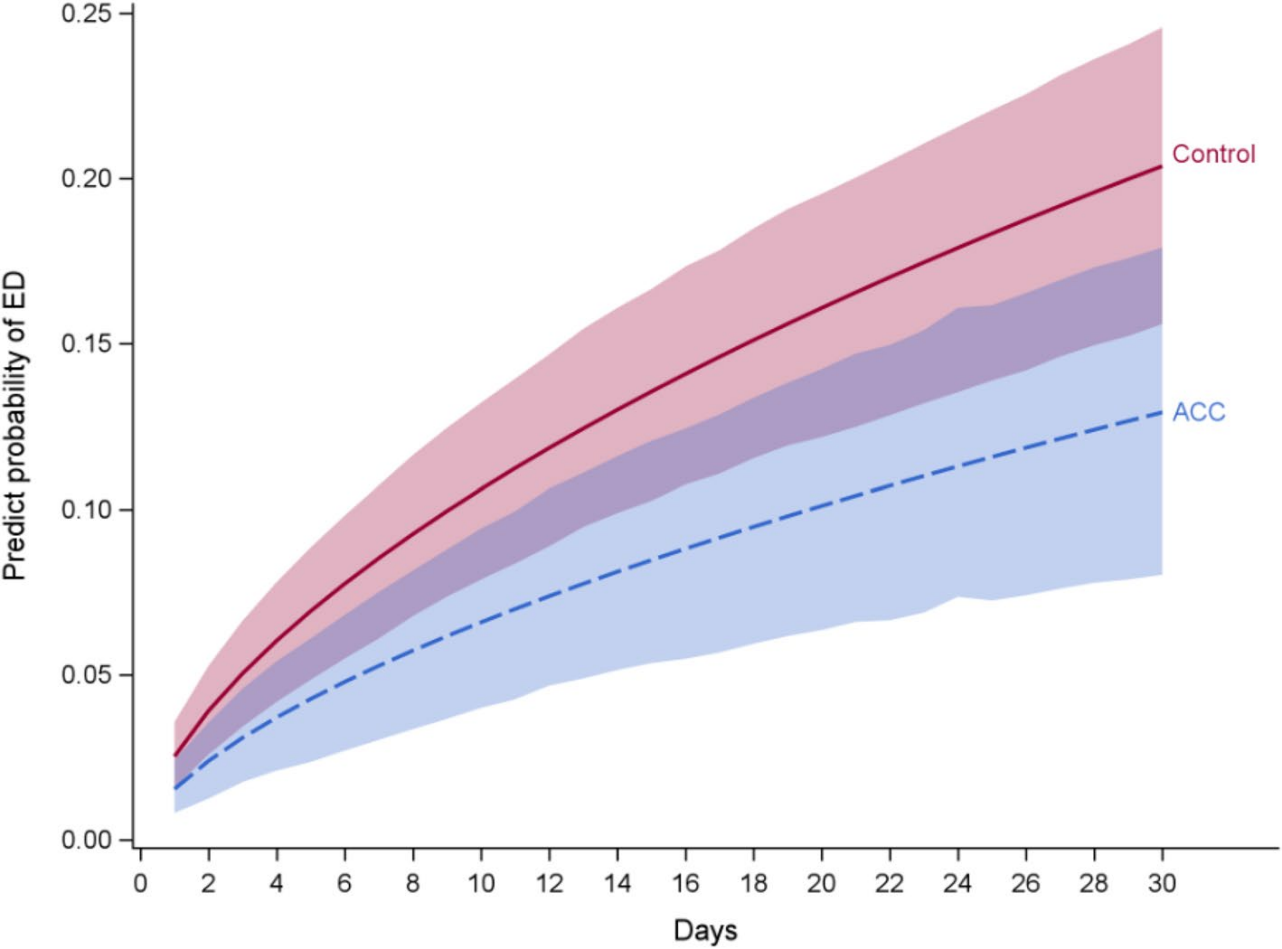
Probability of emergency department visits within 30-days post-discharge

Of the eligible Veterans (*N* = 460) who had a non-VA ED visit between 4/10/2018 – 4/1/2020 and met eligibility criteria, 161 Veterans completed all four core components of the intervention. There were 19,771 eligible control Veterans. The study population characteristics and most of the Elixhauser comorbidity index variables were significantly different prior to matching (Table 1). After matching, there were no significant differences between ACC and control patients. We identified patient factor data and Elixhauser comorbidity data for patients who were eligible for ACC but did not complete the intervention (Table 2). There were substantial differences between this group and the ACC group. Within patients who did not finish the intervention there were more Black patients and more patients located in rural or highly rural areas. Compared to ACC group, this group of patients also had a higher proportion in 19 out of 29 total comorbidity variables, such as hypertension, heart failure, pulmonary disease, diabetes with chronic complications, renal failure, weight loss, depression, etc. When compared to a matched control group ACC had significantly lower unadjusted 30-day ED visit rates. After adjusted for Elixhauser score and prior one year ED visit, ACC also had significantly lower risk to have an ED visit within 30-days of discharge compared to the control group (HR = 0.61, 95% CI = (0.42, 0.92)) (Fig. 1: Probability of emergency department visits within 30-days post-discharge). The control group had higher probability to have a PCP visit within 12-days of discharge, while ACC group had higher probability to have a PCP visit at 13–30 days within discharge (HR = 1.5, 95% CI = (1.01, 2.22)). Both effects were statistically significant. The ACC group showed lower risk on 30-day hospital readmissions although this was not significant. There were no significant differences between ACC and the control group on their 120-day pre-post intervention trend for ED visits, hospitalizations, and PCP visits. The control group had an increasing trend pre-post intervention for 120-day admissions and ACC had a decreasing pre-post intervention trend. This difference was not significant (*p* = 0.09). See Tables 3 and 4 for more details.

**Table 1:** Comparison of control and Advanced Care Coordination groups post-matching

Patient Factors		Control (*N* = 475)	ACC (*N* = 161)	*p*-value
Age (Mean (SD))		63.90 (15.91)	63.47 (15.97)	0.77
Sex = Female(%)		49 (10.3)	16 (9.9)	1.00
**Race (%)**				0.99
	White	389 (81.9)	132 (82.0)	
	Black	57 (12.0)	19 (11.8)	
	Other	11 (2.3)	4 (2.5)	
	Unknown	18 (3.8)	6 (3.7)	
**Urban Rural Highly Rural (%)**				1.00
	Urban	379 (79.8)	129 (80.1)	
	Rural	96 (20.2)	32 (19.9)	
	Highly Rural	0 (0.0)	0 (0.0)	
**Site (%)**				1.00
	Denver	201 (42.3)	67 (41.6)	
	Omaha	274 (57.7)	94 (58.4)	
**Elixhauser Comorbidity Index**				
Hypertension = 1(%)		291 (61.3)	100 (62.1)	0.92
Congestive Heart Failure = 1(%)		53 (11.2)	18 (11.2)	1.00
Chronic Pulmonary Disease = 1(%)		145 (30.5)	48 (29.8)	0.94
Diabetes without Chronic Complications = 1(%)		127 (26.7)	42 (26.1)	0.95
Diabetes with Chronic Complications = 1 (%)		114 (24.0)	36 (22.4)	0.75
Renal Failure = 1 (%)		71 (14.9)	25 (15.5)	0.96
Obesity = 1(%)		86 (18.1)	27 (16.8)	0.79
Weight Loss = 1(%)		27 (5.7)	11 (6.8)	0.74
Fluid and Electrolyte Disorders = 1(%)		105 (22.1)	41 (25.5)	0.44
AIDS/HIV = 1(%)		0 (0.0)	0 (0.0)	1.00
Alcohol Abuse = 1 (%)		62 (13.1)	24 (14.9)	0.65
Anemia Deficiency = 1(%)		91 (19.2)	33 (20.5)	0.80
Rheumatoid Arthritis = 1(%)		13 (2.7)	4 (2.5)	1.00
Blood Loss Anemia = 1 (%)		8 (1.7)	2 (1.2)	0.98
Coagulopathy = 1 (%)		31 (6.5)	10 (6.2)	1.00
Depression = 1(%)		120 (25.3)	28 (23.6)	0.75
Drug Abuse = 1(%)		37 (7.8)	13 (8.1)	1.00
Hypothyroidism = 1(%)		52 (10.9)	19 (11.8)	Hypothyroidism = 1(%)
Liver Disease = 1(%)		69 (14.5)	27 (16.8)	0.58
Lymphoma = 1(%)		5 (1.1)	4 (2.5)	0.35
Metastatic Cancer = 1(%)		11 (2.3)	3 (1.9)	0.98
Other Neurological Disorders = 1(%)		135 (28.4)	48 (29.8)	0.81
Paralysis = 1(%)		28 (5.9)	12 (7.5)	0.61
Peripheral Vascular Disease = 1(%)		74 (15.6)	26 (16.1)	0.96
Psychiatric Disorders = 1(%)		36 (7.6)	15 (9.3)	0.59
Pulmonary Circulation Disorder = 1(%)		6 (1.3)	2 (1.2)	1.00
Solid Tumor without Metastasis = 1 (%)		24 (5.1)	12 (7.5)	0.35
Peptic Ulcer Disease = 1(%)		2 (0.4)	1 (0.6)	1.00
Valvular Disease = 1(%)		42 (8.8)	17 (10.6)	0.62
**Outcomes Prior Intervention**				
1 year Prior PCP visit (mean (SD))		2.65 (2.43)	2.80 (2.30)	0.50
1 year Prior Hospitalization (mean (SD))		0.36 (0.73)	0.37 (0.70)	0.87
1 year Prior ED visit (mean (SD))		1.54 (2.65)	1.63 (2.21)	0.70
**Outcomes Post Intervention**				
30-day Mortality = 1 (%)		5 (1.1)	2 (1.2)	1.00
60-day Mortality = 1 (%)		10 (2.1)	3 (1.9)	1.00
90-day Mortality = 1 (%)		17 (3.6)	5 (3.1)	0.97
30-day ED Visit = 1 (%)		127 (26.7)	29 (18.0)	0.03
60-day ED Visit = 1 (%)		153 (32.2)	45 (28.0)	0.36
90-day ED Visit = 1 (%)		176 (37.1)	54 (33.5)	0.48
30-day Readmission = 1 (%)		21 (4.4)	4 (2.5)	0.39
60-day Hospital Readmission = 1 (%)		36 (7.6)	10 (6.2)	0.69
90-day Hospital Readmission = 1 (%)		44 (9.3)	15 (9.3)	1.00
14-day PCP visit = 1 (%)		140 (29.5)	53 (32.9)	0.47
30-day PCP Visit = 1 (%)		199 (41.9)	80 (49.7)	0.10

*ACC* Advanced Care Coordination, *SD* Standard Deviation, *ED* Emergency Department, *PCP* Primary Care Physician

**Table 2:** Characteristics of eligible patients who did not complete ACC compared to ACC intervention group

	**Eligible Patients Who Did Not Complete ACC Intervention**	**ACC**
	(*N* = 299)	(*N* = 161)
**Patient Factors**		
Age (Mean (SD))	65.42 (13.57)	63.47 (15.97)
Sex = Female (%)	16 (5.4)	16 (9.9)
**Race** (%)		
White	224 (74.9)	132 (82.0)
Black	56 (18.7)	19 (11.8)
Other	6 (2.0)	4 (2.5)
Unknown	13 (4.3)	6 (3.7)
**Urban, Rural, Highly Rural (%)**		
Urban	208 (69.6)	129 (80.1)
Rural	81 (27.1)	32 (19.9)
Highly Rural	10 (3.3)	0 (0.0)
**Elixhauser Comorbidity Index**		
Hypertension = 1(%)	221 (73.9)	100 (62.1)
Congestive Heart Failure = 1(%)	87 (29.1)	18 (11.2)
Chronic Pulmonary Disease = 1(%)	131 (43.8)	48 (29.8)
Diabetes without Chronic Complication = 1(%)	86 (28.8)	42 (26.1)
Diabetes with Chronic Complications = 1(%)	94 (31.4)	36 (22.4)
Renal Failure = 1(%)	69 (23.1)	25 (15.5)
Obesity = 1(%)	65 (21.7)	27 (16.8)
Weight Loss = 1(%)	58 (19.4)	11 (6.8)
Fluid and Electrolyte Disorders = 1 (%)	132 (44.1)	41 (25.5)
AIDS/HIV = 1(%)	2 (0.7)	0 (0.0)
Alcohol Abuse = 1 (%)	78 (26.1)	24 (14.9)
Anemia Deficiency = 1(%)	100 (33.4)	33 (20.5)
Rheumatoid Arthritis = 1(%)	12 (4.0)	4 (2.5)
Blood Loss Anemia = 1 (%)	14 (4.7)	2 (1.2)
Coagulopathy = 1 (%)	50 (16.7)	10 (6.2)
Depression = 1(%)	105 (35.1)	38 (23.6)
Drug Abuse = 1(%)	60 (20.1)	13 (8.1)
Hypothyroidism = 1(%)	50 (16.7)	19 (11.8)
Liver Disease = 1(%)	57 (19.1)	27 (16.8)
Lymphoma = 1(%)	10 (3.3)	4 (2.5)
Metastatic Cancer = 1(%)	15 (5.0)	3 (1.9)
Other Neurological Disorders = 1(%)	123 (41.1)	48 (29.8)
Paralysis = 1(%)	24 (8.0)	12 (7.5)
Peripheral Vascular Disease = 1(%)	74 (24.7)	26 (16.1)
Psychiatric Disorders = 1(%)	77 (25.8)	15 (9.3)
Pulmonary Circulation Disorder = 1(%)	21 (7.0)	2 (1.2)
Solid Tumor without Metastasis = 1(%)	48 (16.1)	12 (7.5)
Peptic Ulcer Disease = 1(%)	10 (3.3)	1 (0.6)
Valvular Disease = 1(%)	68 (22.7)	17 (10.6)

*ACC* Advanced Care Coordination, *SD* Standard Deviation

**Table 3:** Estimated risk of outcomes between intervention and control groups

Outcomes	Hazard Ratio	95% Confidence Interval
30-day ED visit	0.61	0.42, 0.92
30-day Readmission	0.54	0.17, 1.57
PCP visit at 1–12 days	0.61	0.43, 0.88
PCP visit at 13–30 days	1.50	1.01, 2.22
90-day ED visit	0.83	0.61, 1.13
90-day Readmission	1.04	0.58, 1.90
14-day PCP visit	Risk Ratio = 0.94	0.84, 1.05

*ED* Emergency Department, *PCP* Primary Care PhysicianThe hazard ratio shows the estimated ratio of risk in *ED* visits/*PCP* visits/readmissions between the *ACC* intervention and control group. Results indicate that the intervention group had significantly lower risk of having an *ED* visit within 30-days of discharge when compared to the control group

**Table 4:** Estimated Difference-In-Difference for 120-day pre/post discharge for medical care for ACC compared to control group

Outcomes	Difference-In-Difference Estimate	95% Confidence Interval	*p*-value
Number of ED visit	1.05	0.84, 1.31	0.66
Number of Hospitalization	0.84	0.69, 1.03	0.09
Number of PCP visit	1.02	0.87, 1.19	0.79

*ACC* Advanced Care Coordination, *ED* Emergency Department, *PCP* Primary Care Physician

When compared to a control group, for the number of *ED* visits, hospitalizations, and *PCP* visits there are no significant differences in the results between the *ACC* intervention group and the control group on their pre/post intervention trend. However, for the 120-day number of hospitalizations the control group had an increasing trend pre/post intervention and the *ACC* intervention group had a decreasing pre/post intervention trend. This difference in trend was not significant (*p* = 0.09)

**Figure 2:**
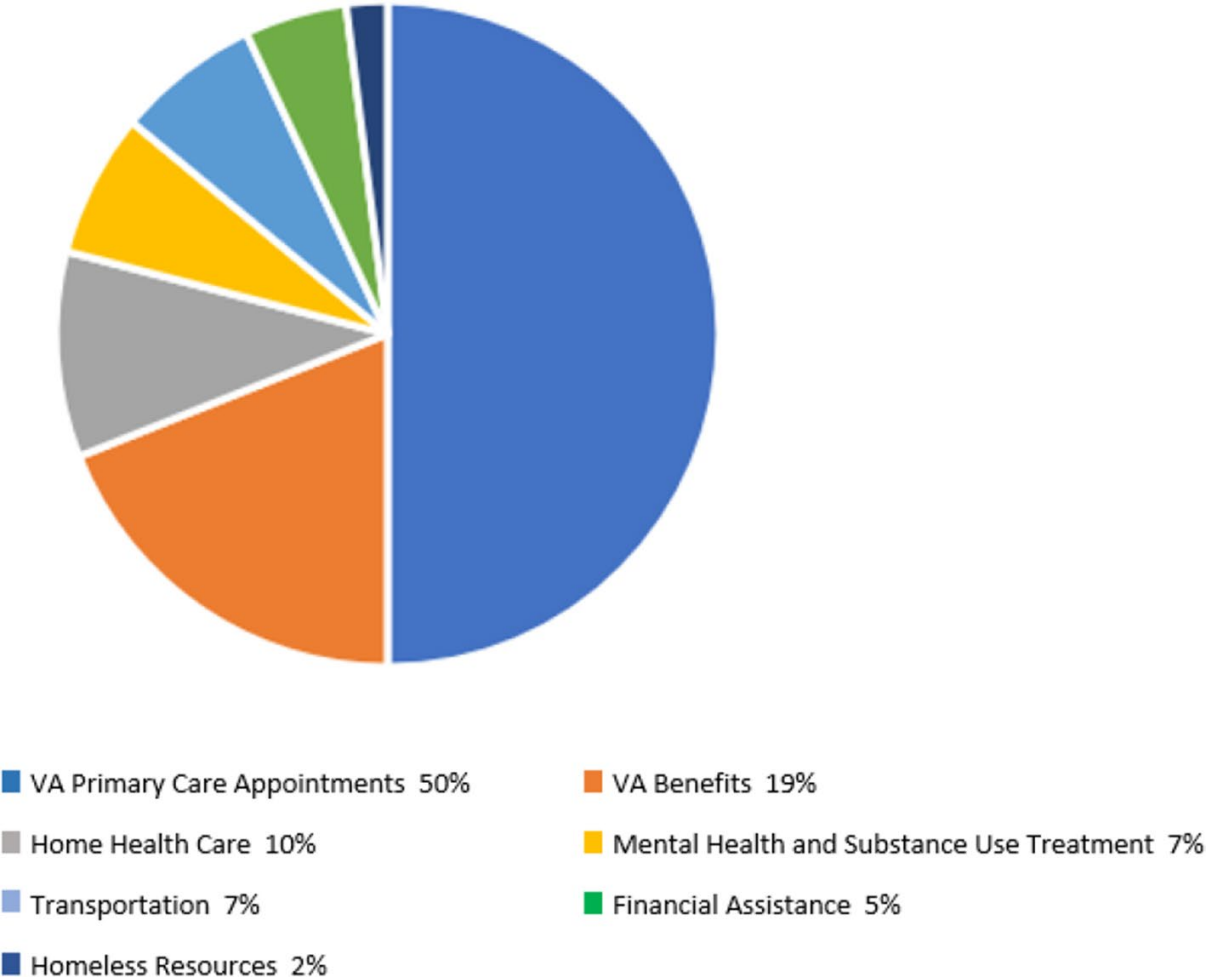
Resources utilized to address dual-use veterans’ social determinants of health

The ACC social workers connected the Veterans who completed all four core components (*N* = 161) to resources addressing SDOH (Fig. 2: Resources utilized to address dual-use veterans’ social determinants of health). Of those resources, 50% were VA PCP appointments, 19% were VA benefits, 10% were home health care, 7% were mental health and substance use treatment, 7% were transportation resources, 5% were financial assistance, and 2% were homeless resources. Through linkage to these resources, the ACC social workers addressed SDOH including access to health care (e.g., PCP appointments, mental health and substance use treatment, home health care, dental assistance, etc.) and economic concerns (e.g., applications for financial benefits and Medicaid, transportation resources including Medicaid, Medicare, and VA transportation, utility/rental assistance, housing vouchers, homeless resources, etc.).

ACC social workers documented adaptations, or any changes to the program core components, program delivery or implementation strategies, throughout the program implementation as they were taking place. There were no adaptations to the program core components, ensuring the fidelity to the original program goals across the settings. However, the program delivery was adapted to fit within local contexts, processes, and stakeholder preferences.

## Discussion

### Summary

We developed and implemented a program addressing dual-use Veteran’s needs. ACC bridged gaps in care coordination for Veterans who utilized non-VA EDs and returned to the VA for care and addressed SDOH for these dual-use Veterans. ACC was initially implemented at ECHCS and disseminated to NWIHCS. When we implemented ACC we reduced the risk of 30-day ED readmissions for dual-use Veterans and connected them to their VA PCP 13–30 days post ED discharge. By addressing SDOH, dual-use Veterans were linked to necessary social and medical resources (e.g., financial resources, medical appointments, etc.).

### Implications of Findings

Results of other studies show care coordination benefits for ED users are mixed [35–37]. Some studies show care coordination does not reduce ED visits and others indicate that care coordination tailored to patients’ complex needs effectively reduces ED utilization since patients are linked to necessary resources [11, 38–42]. Interventions addressing SDOH improved health outcomes and/or reduced health care spending by reducing ED visits [43]. Similarly, our study showed that utilizing a social worker to address SDOH post-ED discharge decreased 30-day ED visits and linked Veterans to essential social and medical resources. Veterans enrolled in ACC had a higher probability of having a PCP visit within 13–30 days post-ED discharge, whereas the control group had a higher probability of a PCP visit within 12-days of discharge. This may be due to ACC’s longitudinal nature where the immediate focus post-ED discharge was on Veterans’ complex SDOH needs and linking them to necessary resources immediately which may not have included a PCP visit during the initial post-ED discharge period. During the initial post-ED discharge period the ACC social workers focused on addressing enrolled Veterans most pressing SDOH needs (e.g., financial resources, VA benefits, etc.) and linked Veterans back to their VA PCP later in the ACC intervention. ACC’s fourth core component was a warm hand-off to the VA PCP, which could be another factor why PCP visits occurred later in the intervention group [44].

### Strengths and Limitations

Our program had many strengths. ACC enhanced care coordination, addressed SDOH for dual-use Veterans, and decreased the risk of 30-day non-VA ED use post-discharge. We developed a training toolkit to ensure fidelity when ACC expanded. Additionally, when we disseminated ACC to NWIHCS it was implemented successfully with fidelity to the program core components; thus, this intervention can be implemented at other VAs indicating generalizability within the VA system. Our team’s multidisciplinary approach and diverse expertise enabled effective implementation and evaluation of ACC.We encountered limitations for our study. There was lack of generalizability as we implemented ACC at two VAs. There could have been other factors, besides the ACC intervention, that caused positive outcomes in the intervention group as this was not a randomized control trial (RCT). Although there could have been selection bias we utilized propensity matching to mitigate this limitation and to serve as an alternative method to a RCT. ACC was designed to assist Veterans who were discharged home from non-VA EDs, were enrolled in ECHCS and NWIHCS, and were not already receiving case management in the VA. There were Veterans who were eligible to participate in ACC, but they did not complete the program. As indicated in Table 2 these patients had differences that may have impacted their health and health disparities (e.g. a higher percentage of patients were Black, lived in rural or highly rural areas, had a higher proportion in 19 out of 29 total comorbidity variables, etc.) from the intervention group. Due to these differences our results may have some selection bias. Future implementation of ACC or similar programs could focus on health equity and inclusion by including telehealth visits for Veterans in rural or highly rural areas and specifically targeting Veterans from various ethnic and racial groups. Veterans with higher proportion of comorbidity variables may be included if future programs include those who are already receiving case management through other programs at the VA, or by reducing exclusion criteria described previously.

## Conclusion

Utilizing a social worker to address SDOH and connect dual-use Veterans to social and medical resources led to a reduction in risk of ED visits in the future when compared to a control group. ACC has been uploaded to the VA’s Diffusion Marketplace (Diffusion Marketplace_ACC Program) to provide education and practical information on how to implement ACC and to promote implementation of similar programs. The site is designed to help organically spread important practices throughout the VA. VAs interested in implementing ACC will have access to our training materials and toolkits. VA providers we collaborated with expressed that ACC was invaluable and have strategized ways to continue our patient-centered approach to care coordination with non-VA hospitals. Following our grant funding period for ACC, ACC program components and approach were integrated into the NWIHCS Office of Community Care to continue addressing dual-use Veterans’ SDOH. Additionally, ACC is one of the identified solutions by the VA nationwide to address high utilization of non-VA EDs. Education on ACC components as well as training materials and resources have been presented to national committees and VAs across the nation who have been identified as having the highest non-VA ED utilization. If selected as a solution, ACC would become a standard of practice funded by and integrated into the VA. Continued implementation of evidence-based interventions for best practices addressing SDOH and care coordination across health care systems is recommended. Future studies should be expanded to focus on Veterans who have more social and medical complex needs to understand the impact of social worker facilitated care coordination.

## Supplementary Information

**Additional file 1.** Denver VA Center of Innovation/Mental Illness Research Education and Clinical Center Veteran Research Engagement Board – Investigator Presentations. This file format is a Microsoft Word Document and the file extension is .doc. The data contained in this file consists of information to be completed and provided to the VREB prior to meeting with them.

**Additional file 2.** Social Work Comprehensive Assessment. This file format is an Adobe PDF and the file extension is .pdf. The data contained in this file consist of questions the social workers asked Veterans to screen them for social (SDOH) and medical needs and to enroll them into the ACC program.

## Data Availability

The datasets generated and or analyzed during the current study are not publicly available due to identifying nature of patients and providers. Furthermore, the VA claims data has patient data that is not to be shared publicly. However, how data was collected and managed during the study are available from the corresponding author on reasonable request.

## Abbreviations

VA: Veteran’s Health Administration
SDOH: Social Determinants of Health
ACC: Advanced Care Coordination
ED: Emergency Department
PCP: Primary Care Physician
HR: Hazard Ratio
CI: Confidence Interval
MISSION: Maintaining Systems and Strengthening Integrated Outside Networks
QI: Quality Improvement
CHTP: Community Hospital Transitions Program
ECHCS: Eastern Colorado Health Care System
VREB: Veteran Research Engagement Board
CoC: Continuity of Care
PACT: Patient Aligned Care Team
NWIHCS: Nebraska-Western Iowa Health Care System
HIPAA: Health Insurance Portability and Accountability Act
VA OCC: VA Office of Community Care
DID: Difference-In-Difference
RCT: Randomized Control Trial
ORH: Office of Rural Health
U.S.: United States
OVAC: Office of Veterans Access to Care
