# Supplementary material for: Optimizing care coordination to address social determinants of health needs for dual-use veterans
Source: BMC Health Serv Res. 2022 Jan 12;22:59. doi: 10.1186/s12913-021-07408-x (PMC8754195; doi:10.1186/s12913-021-07408-x)
Supplement: Supplementary file 1 — Additional file 1. Denver VA Center of Innovation/Mental Illness Research Education and Clinical Center Veteran Research Engagement Board – Investigator Presentations. This file format is a Microsoft Word Document and the file extension is .doc. The data contained in this file consists of information to be completed and provided to the VREB prior to meeting with them. [file 12913_2021_7408_MOESM1_ESM.docx]

Investigator/Lead Contact Name:

COIN or MIRECC (circle one)

Study/Project Name:

Please provide the following research materials for review at least **1 month prior** to your scheduled meeting with the Board:

1. Abstract in plain English or Objective of Project:
2. Attach grant proposal and IRB protocol (if applicable) or other project documents
3. Attach the 5-7 slide presentation (including relevant information about the health topic at hand and one slide with a simple, visual representation of your study design if relevant) that you will use to guide your verbal presentation to the Board.
4. Where on the below Community Engagement Continuum would you like your interaction meeting interaction to be:
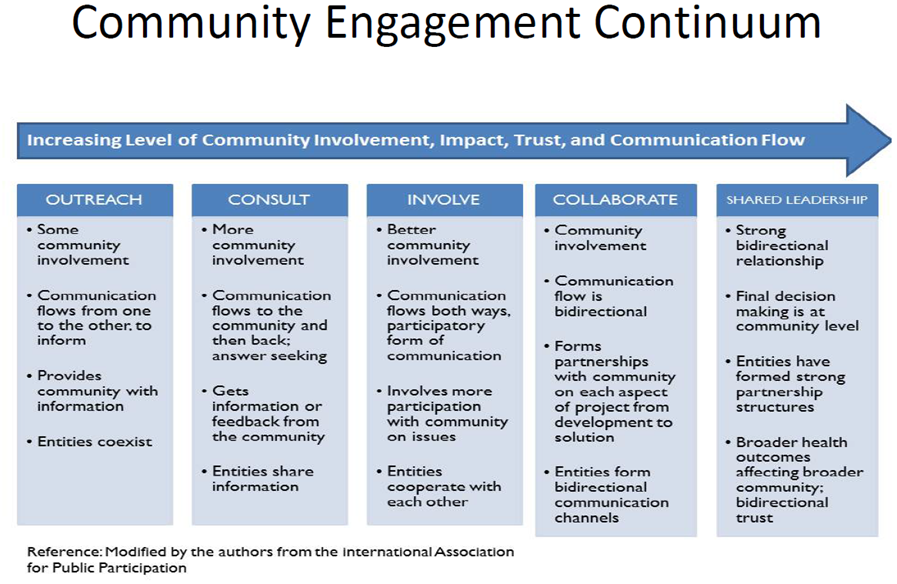
*Examples can be provided by Leah or Kelty. Please contact if you would like more information on this question.
5. What below areas of the project would you like to be informed or impacted by the board engagement? please circle all that apply:
6. 2-3 specific questions for the board that you would like feedback/help on:
